# Methotrexate-Induced Leukoencephalopathy as a Clinical and Radiological Mimicker of Acute Ischemic Stroke Leading to Thrombolysis

**DOI:** 10.7759/cureus.51542

**Published:** 2024-01-02

**Authors:** Vishal V Panicker, Sunesh E Radhakrishnan, Gigy V Kuruttukulam, Jithin A Bose, TT Favas

**Affiliations:** 1 Neurology, Rajagiri Hospital, Kochi, IND

**Keywords:** b-cell acute lymphoblastic leukemia, ischemic stroke, intravenous thrombolysis, stroke mimic, methotrexate-induced leukoencephalopathy

## Abstract

Methotrexate (MTX) is used to treat acute lymphoblastic leukemia (ALL). It acts by inhibiting cell proliferation through its role as a folate antagonist. Despite its positive impact on patients’ survival, high-dose MTX therapy carries risks, notably neurotoxic side effects such as subacute leukoencephalopathy that can mimic stroke symptoms. Recognizing and managing MTX-induced neurotoxicity promptly is crucial. We present a case involving an 18-year-old male diagnosed with B-cell ALL who presented with symptoms of MTX-induced leukoencephalopathy, initially resembling a stroke. The initial neurological examination and imaging results closely resembled those of a stroke, prompting the activation of a stroke code. Due to uncertainty regarding whether it was an acute ischemic stroke, the patient underwent thrombolysis. However, a thorough assessment of the medical history, treatment timeline, and imaging features, combined with the absence of large vessel occlusions on the magnetic resonance angiogram, led to the diagnosis of MTX-induced leukoencephalopathy. Our patient demonstrated complete clinical and radiological improvement within the following ten days. This underscores the significance of thorough history-taking, especially regarding drug history, to distinguish stroke mimics and contemplate MTX-induced leukoencephalopathy as a potential factor in ALL patients receiving MTX treatment. Recognizing these cases is essential to preventing unnecessary thrombolysis.

## Introduction

Methotrexate (MTX) is used in the treatment of acute lymphoblastic leukemia (ALL) to inhibit cell proliferation by acting as a folate antagonist [1,2]. While MTX has significantly improved the survival rates of ALL patients, it is not without its risks. High-dose MTX (HD MTX) therapy can lead to various toxic side effects, including neurotoxicity. Neurotoxicity associated with MTX can present as acute, subacute, or chronic symptoms. Of particular interest is the subacute leukoencephalopathy caused by MTX, which can mimic the clinical and radiological manifestations of a stroke [3]. This complication typically occurs within two weeks of MTX administration and resolves within one week. Symptoms such as weakness of one side of the body, dysarthria, facial droop, headaches, and seizures can resemble those observed in a stroke [4]. Radiological imaging, specifically magnetic resonance imaging (MRI), plays a crucial role in identifying the characteristic radiological manifestations of MTX-induced leukoencephalopathy. Diffusion restriction on diffusion-weighted imaging (DWI) and transient high signal on T2-weighted and FLAIR sequences in the centrum semiovale are suggestive of this condition [4]. It is vital to promptly recognize and manage MTX-induced neurotoxicity to minimize the risk of long-term neurological complications. This article highlights a case involving a young patient who developed MTX-induced subacute leukoencephalopathy, mimicking stroke and prompting the administration of a thrombolytic agent.

## Case presentation

**Figure 1:**
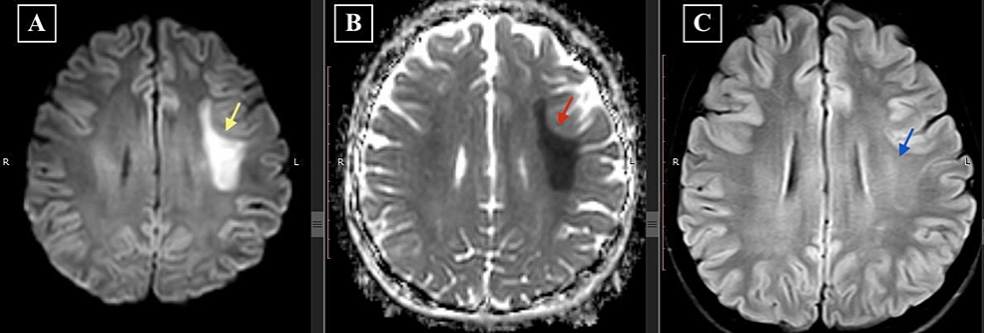
MRI brain on the day of onset of symptoms (A) Diffusion-weighted MRI image showing an area of restricted diffusion in the left frontal centrum semiovale and corona radiata (yellow arrow). (B) The area of restricted diffusion is hypointense on the ADC map (red arrow). (C) FLAIR image showing minimal hyperintensity in the same region (blue arrow). MRI, magnetic resonance imaging

**Figure 2:**
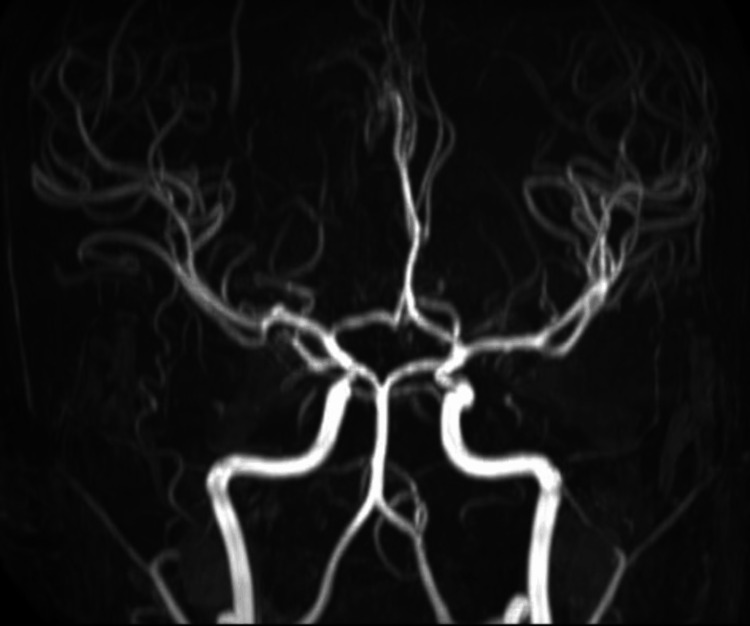
Normal MR angiogram brain

An 18-year-old male diagnosed with B-cell ALL was undergoing chemotherapy (Berlin-Frankfurt-Münster pediatric intermediate-risk regimen). During Induction Phase A, he was treated with prednisone, vincristine, daunorubicin, L-asparaginase, and intrathecal MTX (IT-MTX). Throughout this phase, the patient received three doses of IT-MTX, each at 12 mg. His chemotherapy cycles were uneventful. In Induction Phase B, the regimen included cyclophosphamide, cytarabine, 6-mercaptopurine, and IT-MTX (12 mg), with a planned total of two doses of IT-MTX at 12 mg each. Twelve days after the initial dose of IT-MTX in Induction Phase B (he completed a total of four doses of IT-MTX), the patient reported sudden-onset weakness in the right upper and lower limbs, accompanied by slurred speech, at approximately 11 p.m. The stroke code was activated according to hospital protocol. Neurological examination revealed a GCS score of E4V5M6, right-sided upper motor neuron facial palsy, MRC (Medical Research Council) grade 3/5 power in the right upper and lower limbs, and extensor plantar response on the right. Urgent MRI brain imaging showed diffusion restriction in the left frontal centrum semiovale and corona radiata, with minimal hyperintensity on FLAIR images (Figures 1A-1C). A non-contrast MR angiogram ruled out large vessel occlusions (Figure 2). Considering the potential for an acute ischemic stroke and the patient presenting within the one-hour thrombolysis window, the patient underwent immediate thrombolysis with 0.25 mg/kg of intravenous tenecteplase. The timing of the symptoms, along with the patient's medical history and the recent administration of MTX therapy, contributed to the retrospective diagnosis of MTX-induced leukoencephalopathy. The patient received a 14-day course of leucovorin at a dosage of 5 mg/m^2^ twice daily. Additionally, a five-day regimen of intravenous methylprednisolone was administered at a daily dosage of 500 mg, and a seven-day course of intravenous aminophylline was given at 5 mg/kg every six hours.

Evaluation for other causes of young-onset ischemic stroke, including electrocardiogram, 2D echocardiography, transcranial Doppler study, Holter monitoring, ANA, dsDNA, thrombophilia profile, lupus anticoagulant, blood sugar levels, lipid profile, and homocysteine levels, was normal. A lumbar puncture was performed, and the analysis of cerebrospinal fluid revealed normal values for pressure, cell count, protein levels, and glucose concentration.

**Figure 3:**
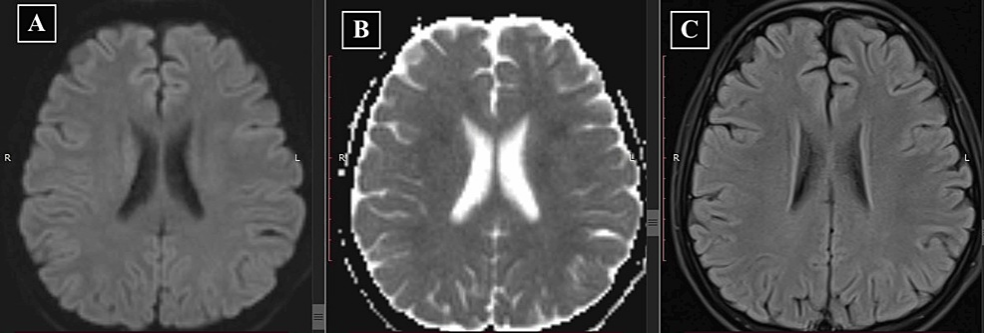
Follow-up MRI scan after one month (A) Normal diffusion-weighted MRI image. (B) Normal ADC map. (c) Normal FLAIR image. MRI, magnetic resonance imaging

The patient's neurological status was closely monitored, and follow-up imaging studies (after seven days and one month) were conducted to evaluate the evolution and/or resolution of symptoms and MRI lesions. Remarkably, his power completely recovered approximately ten days after symptom onset. Follow-up imaging studies after one month showed full resolution of diffusion restriction and FLAIR changes on MRI (Figures 3A-3C). The patient was closely monitored for any recurrence or long-term neurological sequelae. The patient was restarted on IT-MTX after 15 days, and notably, there were no further episodes of a similar nature during the six-month follow-up period.

## Discussion

MTX is a medication that acts as a folate antagonist, inhibiting enzymes involved in nucleotide biosynthesis. This inhibition reduces DNA synthesis, repair, and cell proliferation, ultimately leading to cell death [1]. In the treatment of ALL, HD MTX and/or IT-MTX with leucovorin rescue are commonly used [2,3]. MTX is administered during different stages of ALL treatment in childhood, including induction, consolidation, and maintenance therapy [5]. Advancements in MTX treatment regimens over the years have contributed to significant improvements in the survival rates of ALL patients [6].

However, the use of HD MTX and/or IT-MTX can be associated with various toxic side effects. These include myelosuppression, mucositis, nephrotoxicity, hepatotoxicity, and neurotoxicity [7]. Neurotoxicity from MTX can manifest as acute, subacute, or chronic symptoms. Acute toxicity is usually temporary and does not result in permanent harm. The cumulative incidence of acute encephalopathy after HD MTX administration is around 3% [8]. Common symptoms include somnolence, confusion, headache, vomiting, and seizures, resembling features of meningitis [9]. Subacute and chronic toxicity, on the other hand, involves progressive changes in the brain and/or spinal cord, which can lead to coma and, in severe cases, death. It is believed that MTX induces direct neurotoxic effects, causing damage to neuronal tissues. Additionally, MTX interferes with various metabolic pathways, including folates, excitatory amino acids, homocysteine, S-adenosylmethionine/S-adenosylhomocysteine, adenosine, and biopterins. These biochemical changes contribute to the development of neurotoxic symptoms. Acute toxicity may be partially mediated by adenosine, while subacute and chronic toxicity may involve homocysteine, S-adenosylmethionine/S-adenosylhomocysteine, excitatory amino acids, and biopterins [4,10]. It is important to monitor patients receiving HD MTX for potential neurotoxicity and promptly manage any adverse effects that may arise. Regular assessment and appropriate supportive care can help mitigate the risk of long-term neurological complications.

Subacute leukoencephalopathy is a rare complication that can occur in ALL patients following HD MTX and/or IT-MTX. It typically manifests within two weeks of receiving a high dose or intrathecal administration of MTX and usually resolves within one week. The neurological symptoms associated with subacute MTX-induced leukoencephalopathy can resemble those of a stroke, presenting in an acute or hyperacute manner. These symptoms may include headaches, seizures, dysarthria, facial droop, aphasia, and hemiparesis [4].

Radiological manifestations of MTX-induced leukoencephalopathy can be identified using MRI brain imaging. T2-weighted and FLAIR sequences typically show a transient diffuse high signal in the centrum semiovale while initially sparing subcortical U-fibers. These findings may appear unilaterally or bilaterally. Another radiological marker is the presence of diffusion restriction on DWI, indicating cytotoxic edema. Diffusion restriction is commonly observed across multiple vascular territories in the centrum semiovale and can manifest unilaterally, bilaterally, or in an alternating pattern. In the early stages, diffusion restriction may be observed without corresponding changes on MRI FLAIR sequences [4,11,12]. However, it is worth noting that there have been rare case reports of MTX-induced leukoencephalopathy without diffusion restriction [13,14].

In one of the largest cohorts studied of 369 children with ALL, it was found that 13 (3.8%) patients developed clinical neurotoxicity related to MTX treatment. However, when rechallenged with IT-MTX and/or HD MTX, 12 of these patients did not experience a recurrence of neurotoxicity. Additionally, leukoencephalopathy was detected on MRI in 23.3% of all patients and 20.6% of asymptomatic patients. MRI findings in 74% of asymptomatic and 58% of symptomatic patients persisted at the end of therapy [15].

The treatment of MTX-induced leukoencephalopathy remains unclear, and there is limited evidence to support specific interventions. Leucovorin rescue with aminophylline is commonly used and generally well tolerated, which may be considered as a treatment approach [16]. Another treatment modality that has been explored is the administration of dextromethorphan [17]. However, it is challenging to draw definitive conclusions about the efficacy of these treatments due to the lack of control groups, especially considering the transient nature of MTX neurotoxicity and the rapid resolution of symptoms without pharmaceutical intervention in most patients. Further research and controlled studies are needed to better understand the optimal management strategies for MTX-induced leukoencephalopathy.

It is important to note that omitting MTX for central nervous system (CNS)-directed therapy following a neurotoxic episode can increase the risk of CNS relapse. This was demonstrated in a large cohort study involving pediatric patients with ALL. Among 95 patients who experienced MTX neurotoxicity, the five-year CNS relapse-free survival was 89% when IT-MTX was permanently discontinued and replaced with alternate IT-MTX, compared to 95% when patients continued to receive the prescribed doses of IT-MTX as per the treatment protocol. These findings emphasize the importance of continuing optimal therapy to maximize the chances of a cure [18].

Several studies have provided evidence supporting the safety of rechallenging patients with MTX after an episode of neurotoxicity. These studies have shown that a significant majority of patients, ranging from 82% to 92%, did not experience a recurrence of neurotoxic symptoms [15,19]. Rechallenging with MTX should be delayed until complete neurological recovery, which typically occurs within a week. This may result in a slight omission or delay of MTX therapy by one to two weeks. However, in most patients, it is possible to resume standard therapy successfully without the need for significant delays, substitutions, or prophylactic medications.

In our case, we opted for thrombolysis under the suspicion of acute ischemic stroke, a decision that might have been avoidable with a more definitive diagnosis. Our patient showed complete clinical improvement within 10 days. Subsequently, during the six-month follow-up period, upon restarting IT-MTX, there were no recurrent episodes of a similar nature.

## Conclusions

When patients exhibit stroke-like symptoms after receiving high doses of MTX intravenously or intrathecally, it is crucial to consider MTX-induced leukoencephalopathy as a potential cause. It can present with asymmetrical or unilateral diffusion restriction on MRI, which can closely resemble stroke images especially when unilateral. To differentiate it from a stroke, a comprehensive clinical history and information about drug administration, along with imaging, are very important. This holistic approach is instrumental in establishing an accurate diagnosis. Healthcare professionals outside of oncology settings should be particularly vigilant about the possibility of MTX toxicity mimicking a stroke. This awareness is paramount in effectively triaging patients with MTX-induced leukoencephalopathy, as it helps prevent the initiation of stroke treatment protocols such as thrombolysis, which may have adverse effects in these cases.
